# Willis-Ekbom disease is not associated with poor cardiovascular health in adults

**DOI:** 10.1186/s12952-015-0038-4

**Published:** 2015-11-06

**Authors:** Brynn K. Dredla, Oscar H. Del Brutto, Augustine S. Lee, Pablo R. Castillo

**Affiliations:** Department of Neurology, Mayo Clinic, Jacksonville, FL USA; Department of Critical Care Medicine, Mayo Clinic, Jacksonville, FL USA; Division of Pulmonary and Allergy Medicine, Mayo Clinic, 4500 San Pablo Rd, Jacksonville, FL 32224 USA; School of Medicine, Universidad Espíritu Santo, Guayaquil, Ecuador

**Keywords:** Cardiovascular risk, Restless legs syndrome, Sleep

## Abstract

**Background:**

Willis-Ekbom disease (WED), also called *restless legs syndrome* (RLS), is a neurologic sensorimotor disease that may be associated with cardiovascular disease. Given high morbidity and mortality rates of cardiovascular disease worldwide, we assessed the relation between WED/RLS and cardiovascular health risks in a native South American population. We prospectively analyzed data from The Atahualpa Project of Ecuadorian adults aged 40 years and older. Physicians interviewed consented persons on the health behavior and health factors of the American Heart Association (AHA) for ideal cardiovascular health in adults and underwent fasting laboratory blood collection and blood pressure evaluation. Certified neurologists conducted face-to-face interviews using the International Restless Legs Syndrome Study Group (IRLSSG) field instrument. Persons testing positive for WED/RLS and age-and sex-matched controls underwent confirmatory physical examinations conducted by a neurologist and a sleep specialist to whom IRLSSG designation was blinded.

**Findings:**

Of 665 persons, 94 (14 %) tested positive in IRLSSG; 40 (6 %) had a diagnosis of WED/RLS after neurologic examination and interview. Patients with WED/RLS were younger (53.5 vs 59.9 years, *P* = .001), without significant differences in sex ratios. Among AHA risk factors, only obesity was significantly more prevalent among patients with WED/RLS (42.5 % vs 23.5 %, *P* = .01). However, after adjustment for confounders, body mass index was not significantly associated with WED/RLS.

**Conclusions:**

In adult Amerindians, although obesity and body mass index were associated with WED/RLS on univariate analyses, the association was not present after adjustment for confounders. No other significant associations were found between WED/RLS and AHA cardiovascular metrics.

## Background

Restless legs syndrome (RLS), also known as *Willis-Ekbom disease* (WED), is a neurologic sensorimotor disease characterized by an unpleasant urge to move the legs, with symptoms worse at night than during the day [1]. Recent literature has shown the cognitive and physical impacts that WED has had on approximately 10 % of persons within Northern European populations (ie, Italy, Sweden, Finland, and Germany) and the United States [2–6]. However, the association between WED and cardiovascular disease (CVD) continues to be debated. According to the World Health Organization, the number 1 and number 2 most common causes of death from 2000 to 2012 worldwide were secondary to CVD—ischemic heart disease and stroke, respectively. Therefore, recognition and prompt treatment of exacerbating factors are prudent, and first identification of those factors is needed.

Previous epidemiologic evaluations of WED and various health variables have been conducted through self-reported questionnaires or telephone interviews by trained interviewers [2, 3, 6–11]. Current data show varying degrees of association of WED to cardiovascular risk factors, with most studies conducted in European populations. In this regard, data among Native Amerindians are nonexistent. Therefore, we conducted an epidemiologic cross-sectional study to evaluate WED and cardiovascular risk factors in a native Ecuadorian population. Our goal was to determine whether a WED diagnosis, based on the 2003 position paper of the [International Restless Legs Syndrome Study Group (IRLSSG)], has a correlation with any of 7 cardiovascular metrics—smoking, body mass index (BMI), exercise, diet, blood pressure, glucose, and cholesterol—after controlling for age, sex, and alcohol intake. We hypothesized that WED is associated with a poor cardiovascular health status or with at least 1 of the 7 metrics in the “poor” range.

## Methods

### Study population

Atahualpa is a rural costal Ecuadorian village. It is located at sea level, 10 miles east of the Pacific Ocean (2°18′S, 80°46′W). The village receives 12 hours of sunlight daily throughout the year, and its weather is hot and dry with scarce rain. Its inhabitants are phenotypically short statured (mean [SD] height, 148.5 [9.7] cm) and most have abdominal obesity (56.7 % of the adult population have a waist-to-height ratio ≥0.6) [12]. More than 95 % of the inhabitants belong to the Ecuadorian Native/Mestizo ethnic group (Amerindians) [13]. Most men work as carpenters, farmers, or laborers; most women are homemakers [14]. Their diet is rich in fish and carbohydrates and lacks dairy products, polyunsaturated fats, and beef [14]. Data were obtained from 665 adult Amerindians age 40 years or older living in Atahualpa.

### The Atahualpa project and study design

The Atahualpa Project is an epidemiologic, population-based study with the goal to reduce the burden of neurologic and CVDs in persons residing in rural coastal Ecuador [14]. The protocol and informed consent form were approved by the institutional review board of Hospital-Clinica Kennedy, Guayaquil, Ecuador (FWA 00006867). Data collection was performed in 2 phases. Phase 1 consisted of an initial door-to-door survey done by trained rural doctors to assess demographic characteristics of all Atahualpa residents aged 40 years or older and who lived in the community for 3 months before the prevalence day (June 15, 2013) [13]. Consented persons underwent fasting (ie, >12 hours since last meal) laboratory work for glucose and total cholesterol. They were interviewed by rural doctors to ascertain the 7 metrics of health behaviors and health factors set forth by the Goals and Metrics Committee of the Strategic Planning Task Force of the American Heart Association (AHA) for ideal cardiovascular health of adults older than 20 years [15]. For each metric, persons either met the *ideal* criteria as stated or were classified as *poor*.

Neurologists then conducted a face-to-face questionnaire to identify persons with suspected WED [13]. The IRLSSG field instrument developed in 2003 was used because it had been validated in Ecuadorian Spanish-speaking communities [16]. Three of its 4 questions must be answered affirmatively to have suspicion of WED.

Phase 2 consisted of examination by a certified neurologist (O.H.D.B.) and a certified sleep specialist (P.R.C.) of all persons screened for suspected WED (ie, answered “yes” to 3 of 4 questions). In addition, persons who were considered negative for WED during phase 1 also underwent a complete interview and neurologic examination to assess for a possible false-negative result during the survey. For every 1 person in whom WED was suspected, 2 age-and sex-matched persons without WED were evaluated. Whether a person had a suspected case of WED or was in the negative control group was masked to the neurologists, who spoke Spanish and English.

### Statistical analysis

Given adequate samples in each group, univariate comparisons of continuous variables between patients with and without WED were analyzed with *t* test, with assumption of unequal variances. Categorical comparisons between groups were evaluated with Fisher exact test because of limited events in some categories. The ability of WED to predict BMI was tested in a multivariate linear regression model adjusting for age, sex, excessive alcohol consumption (defined as consuming 8 drinks or more per week), smoking, and poor diet. Differences are considered significant when *P* < .05.

## Results

The initial census survey identified 688 Atahualpa residents aged 40 years and older, of whom 23 declined participation [13]. Subsequently, 665 persons underwent face-to-face interviews with administration of the IRLSSG field instrument. Ninety-four persons (14 %) tested positive on the questionnaire, but after expert clinical interview by neurologists and a formal neurologic examination, 40 (6 %) received the diagnosis of WED. Nineteen of the 54 false-positive cases had chronic knee arthritis; 12, nocturnal cramps; 10, painful peripheral neuropathy; 8, peripheral artery disease; and 5, nonspecific concerns not categorizable as a specific WED mimic disorder. None of the 188 control subjects had a diagnosis after the interview and neurologic examination.

Characteristics of the study population are outlined in Table 1 (mean age, 59.5 years). The comparison between the WED population and the controls (Table 2) showed that in univariate comparisons, the WED group was younger and more obese but without significant differences in blood pressures, blood glucose, smoking, or excessive alcohol use. No significant differences were found in frequency of poor diet or exercise between the groups. Therefore, the only risk factor among the 7 AHA cardiovascular metrics that was associated with WED was BMI, where 42.5 % of patients with WED had a poor BMI metric (ie, BMI ≥25 kg/m^2^) vs 23.5 % of those without WED (*P* = .01). However, when adjusted for obesity confounders, the association between WED and BMI was no longer statistically significant (β = 1.25, *P* = .11).

**Table 1:** Characteristics of the 665 initial study participants

Characteristic	Population	
Mean age, y	59.5	
Sex, %		
Male	42	
Female	58	
Daily alcohol intake, No.		
≥50 g	114	
<50 g	551	
Cardiovascular Health Metric	Ideal Rating, %	Poor Rating, %
Blood pressure	63.6	36.4
Exercise	93.8	6.2
Total cholesterol level	88.7	11.3
Fasting glucose concentration	70.4	29.6
BMI	75.3	24.7
Smoking	98.2	1.8
CVH status	30.2	69.8

*Abbreviations*: *BMI* body mass index, *CVH* cardiovascular health

**Table 2:** Comparison of persons with WED/RLS vs without WED/RLS

Characteristic	WED/RLS Status		*P* value
	Study participants (*n* = 40)	Control subjects (*n* = 625)	
Sex, % female	62.5	57.4	.62
Age, mean (SD), y	53.45 (7.9)	59.91 (12.7)	.001^a^
Excessive alcohol use, %	20.0	17.0	.66
Smoking, %	2.5	1.8	.53
Poor exercise, %	5.0	6.24	>.99
Poor diet, %	5.0	3.5	.65
Blood pressure, mean (SD), mm Hg			
Total	133 (22.6)	138 (25.4)	.17
Systolic	79 (11.3)	77 (11.9)	.29
Diastolic	97 (14.1)	97 (14.6)	.87
Cholesterol, mean (SD), mg/dL	198 (30.3)	198 (.8)	.92
BMI, mean (SD)	28 (4.4)	26 (4.8)	.02^a^
Glucose, mean (SD), mg/dL	124 (47)	140 (88)	.05

*Abbreviations*: *BMI* body mass index, *RLS* restless legs syndrome, *WED* willis-ekbom disease

^a^Statistically significant value

## Discussion

Previously, obesity has been associated with an increased risk of WED [17]. In the present study, we found that patients with WED and a high BMI did not have poor cardiovascular health after adjustment for confounders. This lack of association may have been statistically nonsignificant because of power issues, but it supports the notion that increased BMI is not the only determinant of obesity-related complications. The so-called metabolically healthy obese phenotype is thought to confer a lower CVD risk than obesity with the typical associated metabolic changes [18]. Therefore, racial and genetic differences in our native population might account for these results.

Previous European studies have found varied findings. Benediktsdottir et al [19] reported that WED/RLS was not associated with hypertension within an adult population in Iceland and Sweden. Högl et al [5] and Rothdach et al [20] found no association between cardiovascular lifestyle and WED/RLS. Other large population-based studies, such as the Wisconsin Sleep Cohort Study [2] and the Sleep Heart Health Study by Winkelman et al [11], showed an association between WED/RLS frequency and increased prevalence of CVD and coronary artery disease. However, data were collected through self-administered questionnaires, with the majority of such studies conducted in predominantly white populations.

From the cardiovascular data, we know that major CVDs are the leading causes of death among US Hispanic persons; however, data are lacking among minority groups [21]. Innovative cardiovascular prevention strategies are needed in this Amerindian minority group.

## Conclusions

This study showed no correlation between cardiovascular health status and WED. Our analysis of a homogeneous Ecuadorian Amerindian population is unique and adds diversity to the RLS literature.

A weakness of our study is the absence of objective overnight data, such as the periodic limb movement burden during sleep (PLMS) that is frequently present for persons with WED/RLS. Due to increases in blood pressure reported in association with PLMS [22], it is possible that only persons with WED/RLS symptoms and concurrent PLMS have worse cardiovascular health. Nevertheless, our study showed no correlation of WED/RLS symptoms alone with the AHA metrics for ideal cardiovascular health.

Our study’s strength lies within its methodology (2 phases). The face-to-face interview and formal neurologic examination were performed by neurologists to whom study specifics were blinded, to avoid WED mimics influencing data analysis.

Further studies with objective overnight data are needed to assess the relation of WED/RLS (with and without PLMS) with cardiovascular health.

## Abbreviations

AHA: american heart association
BMI: body mass index
CVD: cardiovascular disease
IRLSSG: international restless legs syndrome study group
PLMS: periodic limb improvement burden during sleep
RLS: restless legs syndrome
WED: willis-ekbom disease

## References

[1] International Restless Legs Syndrome Study Group. 2012 revised IRLSSG diagnostic criteria for RLS [Internet]. [cited 2015 Apr 24]. Available from: http://irlssg.org/diagnostic-criteria/

[2] Winkelman JW, Finn L, Young T (2006). Prevalence and correlates of restless legs syndrome symptoms in the Wisconsin Sleep Cohort. Sleep Med.

[3] Juuti AK, Laara E, Rajala U, Laakso M, Harkonen P, Keinanen-Kiukaanniemi S (2010). Prevalence and associated factors of restless legs in a 57-year-old urban population in northern Finland. Acta Neurol Scand.

[4] Moller C, Wetter TC, Koster J, Stiasny-Kolster K (2010). Differential diagnosis of unpleasant sensations in the legs: prevalence of restless legs syndrome in a primary care population. Sleep Med.

[5] Hogl B, Kiechl S, Willeit J, Saletu M, Frauscher B, Seppi K (2005). Restless legs syndrome: a community-based study of prevalence, severity, and risk factors. Neurology.

[6] Wesstrom J, Nilsson S, Sundstrom-Poromaa I, Ulfberg J (2008). Restless legs syndrome among women: prevalence, co-morbidity and possible relationship to menopause. Climacteric.

[7] Ulfberg J, Nystrom B, Carter N, Edling C (2001). Prevalence of restless legs syndrome among men aged 18 to 64 years: an association with somatic disease and neuropsychiatric symptoms. Mov Disord.

[8] Ohayon MM, O’Hara R, Vitiello MV (2012). Epidemiology of restless legs syndrome: a synthesis of the literature. Sleep Med Rev.

[9] Foley D, Ancoli-Israel S, Britz P, Walsh J (2004). Sleep disturbances and chronic disease in older adults: results of the 2003 National Sleep Foundation Sleep in America Survey. J Psychosom Res.

[10] Phillips B, Hening W, Britz P, Mannino D (2006). Prevalence and correlates of restless legs syndrome: results from the 2005 National Sleep Foundation Poll. Chest.

[11] Winkelman JW, Shahar E, Sharief I, Gottlieb DJ (2008). Association of restless legs syndrome and cardiovascular disease in the Sleep Heart Health Study. Neurology.

[12] Del Brutto OH, Santamaria M, Ochoa E, Penaherrera E, Santibanez R, Pow-Chon-Long F (2013). Population-based study of cardiovascular health in Atahualpa, a rural village of coastal Ecuador. Int J Cardiol.

[13] Del Brutto OH, Del Brutto VJ, Zambrano M, Castillo PR (2014). Prevalence of Willis-Ekbom disease in rural coastal Ecuador: a two-phase, door-to-door, population-based survey. J Neurol Sci.

[14] Del Brutto OH, Mera RM, Farfan R, Castillo PR, Atahualpa Project Investigators (2014). Cerebrovascular correlates of sleep disorders: rational and protocol of a door-to-door survey in rural coastal Ecuador. J Stroke Cerebrovasc Dis.

[15] Lloyd-Jones DM, Hong Y, Labarthe D, Mozaffarian D, Appel LJ, Van Horn L (2010). American Heart Association Strategic Planning Task Force and Statistics Committee. Defining and setting national goals for cardiovascular health promotion and disease reduction: the American Heart Association’s strategic impact goal through 2020 and beyond. Circulation.

[16] Castillo PR, Kaplan J, Lin SC, Fredrickson PA, Mahowald MW (2006). Prevalence of restless legs syndrome among native South Americans residing in coastal and mountainous areas. Mayo Clin Proc.

[17] Gao X, Schwarzschild MA, Wang H, Ascherio A (2009). Obesity and restless legs syndrome in men and women. Neurology.

[18] Badoud F, Perreault M, Zulyniak MA, Mutch DM (2015). Molecular insights into the role of white adipose tissue in metabolically unhealthy normal weight and metabolically healthy obese individuals. FASEB J.

[19] Benediktsdottir B, Janson C, Lindberg E, Arnardottir ES, Olafsson I, Cook E (2010). Prevalence of restless legs syndrome among adults in Iceland and Sweden: lung function, comorbidity, ferritin, biomarkers and quality of life. Sleep Med.

[20] Rothdach AJ, Trenkwalder C, Haberstock J, Keil U, Berger K (2000). Prevalence and risk factors of RLS in an elderly population: the MEMO study. Memory and Morbidity in Augsburg Elderly. Neurology.

[21] Daviglus ML, Talavera GA, Aviles-Santa ML, Allison M, Cai J, Criqui MH (2012). Prevalence of major cardiovascular risk factors and cardiovascular diseases among hispanic/latino individuals of diverse backgrounds in the United States. JAMA.

[22] Pennestri MH, Montplaisir J, Colombo R, Lavigne G, Lanfranchi PA (2007). Nocturnal blood pressure changes in patients with restless legs syndrome. Neurology.

